# Investigating the Stability of Cu_2_Se Superionic Thermoelectric Material in Air Atmosphere

**DOI:** 10.3390/ma18174152

**Published:** 2025-09-04

**Authors:** Paweł Nieroda, Małgorzata Rudnik, Marzena Mitoraj-Królikowska, Ewa Drożdż, Dawid Kozień, Juliusz Leszczyński, Andrzej Koleżyński

**Affiliations:** Faculty of Materials Science and Ceramics, AGH University of Krakow, al. A. Mickiewicza 30, 30-059 Krakow, Poland; pnieroda@agh.edu.pl (P.N.); malrud@student.agh.edu.pl (M.R.); mmitoraj@agh.edu.pl (M.M.-K.); edrozdz@agh.edu.pl (E.D.); kozien@agh.edu.pl (D.K.); jleszczy@agh.edu.pl (J.L.)

**Keywords:** copper(I) selenide, thermoelectric materials, spark plasma sintering (SPS), oxidation, superionic thermoelectric

## Abstract

Copper selenide (Cu_2_Se) has garnered significant attention as an exceptional thermoelectric material due to its high thermoelectric figure of merit (*ZT* values > 2). This remarkable efficiency makes it a strong candidate for various applications. However, the practical deployment of thermoelectrics often requires operation in an oxygen-containing atmosphere, which poses a significant challenge for Cu_2_Se due to its environmental instability. This work investigates the environmental behavior of high-purity Cu_2_Se, which was synthesized via a direct high-temperature reaction and spark plasma sintering (SPS). Our Temperature-Programmed Oxidation (TPO) studies determined that the onset of oxidation occurs at a temperature as low as 623 K. Further analysis using SEM–EDS confirmed the formation of copper oxides, Cu_2_O and CuO. Critically, thermogravimetric analysis (TGA) revealed that the SeO_2_ formation and sublimation process is an equally profound degradation mechanism, alongside copper oxidation, particularly within the optimal 673–973 K temperature range. Complementary XRD studies of samples annealed in air underscore this severe material degradation, which is especially devastating between 873 and 973 K. Ironically, this is the precise temperature window where Cu_2_Se’s highest *ZT* values have been reported. Our findings demonstrate that the direct application of Cu_2_Se in air is impractical, highlighting the urgent need for developing robust protective layers to unlock its full potential.

## 1. Introduction

Copper(I) selenide (Cu_2_Se) is a superionic thermoelectric material [1,2,3,4,5,6,7,8] with promising applications in thermoelectric generators (TEGs) [9,10,11,12,13]. The most important material parameter influencing the efficiency of thermoelectric generators is the thermoelectric figure of merit *ZT* = *α*^2^*σκ*^−1^*T*, (where *α*—Seebeck coefficient, *σ*—electrical conductivity, *κ*—thermal conductivity, *T*—temperature) [14,15,16,17], which depends solely on the transport properties of the materials. Cu_2_Se-based thermoelectric materials exhibit very high *ZT* > 2, achieved through doping, e.g., *ZT*_max_ = 2.14 for Cu_1.98_Li_0.02_Se, *T* = 973 K [18], *ZT*_max_ = 2.1 for Cu_1.96_Na_0.04_Se, *T* = 973 K [19], *ZT*_max_ = 2.62 for Cu_1.94_Al_0.02_Se, *T* = 1029 K [20], or by introducing nanoinclusions, e.g., *ZT*_max_ = 2.4 for Cu_2_Se + 0.75 wt% CNTs (carbon nanotubes), *T* = 1000 K [21], *ZT*_max_ = 2.6 for Cu_1.99_Se + 0.9 vol.% B_4_C, *T* = 1025 K [22], *ZT*_max_ = 2.8 for Cu_1.99_Se + 0.25 wt% TiO_2−n_, *T* = 973 K [23], *ZT*_max_ = 2.2 for Cu_2_Se/10 wt% GeTe, *T* = 923 K [24], *ZT*_max_ = 2.7 for Cu_2_Se_1.005_/0.1 mol% BiCuSeO, *T* = 973 K [25]. These high *ZT* values for Cu_2_Se are primarily a result of its very low lattice thermal conductivity, *κ*_l_ = 0.4–0.6 W·m^−1^K^−1^, combined with high electrical conductivity σ ~ 10^4^–10^5^ S·m^−1^ [26]. This phenomenon is explained by the “phonon-liquid electron-crystal” (PLEC) concept [27], which we have described in our earlier works [28,29]. Thermoelectric materials have been successfully used for many years in radioisotope thermoelectric generators (RTGs) for space missions [30,31,32]. However, ongoing research focuses on their terrestrial applications. For example, they are being developed for waste heat recovery in thermoelectric generators mounted on car exhaust pipes [33,34]. These systems aim to recover about 30% of the energy from fuel combustion in internal combustion engines, which is typically lost as waste heat from exhaust gases. Another promising area is waste heat recovery in marine applications [35,36,37]. These applications demand that thermoelectric materials with high *ZT* values are also resistant to degradation in air, ensuring their thermoelectric properties and, consequently, the TEG’s efficiency does not deteriorate. Taskinen et al. [38] investigated the oxidation mechanism of Cu_2_Se at temperatures between 723 and 823 K in a pure oxygen atmosphere. They observed the formation of copper oxides (Cu_2_O and CuO) on the Cu_2_Se surface, alongside the evaporation of selenium as SeO_2_ through the porous oxide layer and its cracks. The authors also pointed to the possibility of intermediate oxidation products like Cu_2_SeO_4_ and CuSeO_3_ [39]. However, it is important to note that the highest *ZT*_max_ values for Cu_2_Se were achieved at approximately 973 K. Therefore, from the perspective of potential Cu_2_Se-based materials for TEG applications, it seems crucial to investigate their stability in a higher temperature range than what was performed in the study by Taskinen et al. [38]. The aim of this work was to investigate the stability of Cu_2_Se in an air atmosphere at temperatures up to 973 K. This was performed to determine its suitability for thermoelectric generator applications within the temperature range where Cu_2_Se-based materials exhibit high *ZT* values. Our findings indicate a lack of stability, particularly in the 873–973 K temperature range. This suggests the necessity of applying protective layers to Cu_2_Se-based materials to prevent degradation in the air. Such protective layers are already used with some other thermoelectric materials, like CoSb_3_ [40,41] and Mg_2_Si [42,43].

## 2. Materials and Methods

The synthesis of Cu_2_Se was carried out in quartz ampoules via a direct reaction between Cu powder (Alfa Aesar, 99.9%) and Se granules (Alfa Aesar, 99.999%). The materials were weighed in a glove box (*p* < 0.5 ppm O_2_ and H_2_O) and reacted at 1423 K for 15 min, followed by 873 K for 7 days. The resulting ingots were ground in an agate mortar and obtained powders sintered in graphite dies using the SPS technique (RACS25-hybrid device from Materials Design Systems and Devices LLC, Kraków, Poland) with AC current to eliminate potential copper ion migration (*T* = 823 K, *t* = 1 min, *p* = 50 MPa, *v* = 100 K·min^−1^, vacuum *p* = 2 × 10^−2^ mbar). The density of the samples was measured using the hydrostatic method and was 6.65 g·cm^−3^. The obtained samples were characterized by *X*-ray powder diffraction (XRD) (*X*-ray Diffractometer Panalytical Empyrean, CuKα, *λ* = 1.5418 Å) and scanning electron microscopy (SEM) (ThermoFisher Scientific Scios 2 with a microscope equipped with an EDAX detector). Temperature-programmed oxidation (TPO_x_) measurements were conducted using a Micromeritics ChemiSorb 2750 apparatus. Samples, prepared as pellets and powders, were loaded into a quartz reactor and heated to 903 K at a rate of 10 K·min^−1^. Oxidation (TPO_x_) processes were carried out under flow of 5% O_2_/Ar mixture. The isothermal thermogravimetric oxidation tests were carried out in a temperature range of 673–973 K in air (a schematic diagram of the experimental setup is shown in Figure S1). The MK2 Vacuum Head CI Electronics Ltd. (Wiltshire, UK) setup, with a capacity of 5 g, was used for the experiments. The setup consists of a microbalance housed on an aluminum block with a metal case mounted on a rigid homemade framework isolated from vibrations. In order to protect microbalance against additional vibrations, the resistance furnace used for heating samples was mounted separately from the thermobalance framework. The samples and counterweight were caught with two rigid lattice arms of thermobalance using special quartz holders and placed in an open quartz tube. An optical position detector and a force coil attached to the arms were employed to maintain balance and produce stable, accurate mass measurements. The mass changes were continuously recorded by means of the CI Disbal control unit with an accuracy of 10^–6^ g, and directly transferred to a personal computer. The CI Disbal control unit was also connected with a *K*-type thermocouple for temperature reading and additional temperature control, separately from the resistance furnace heating control system.

## 3. Results and Discussion

**Figure 1:**
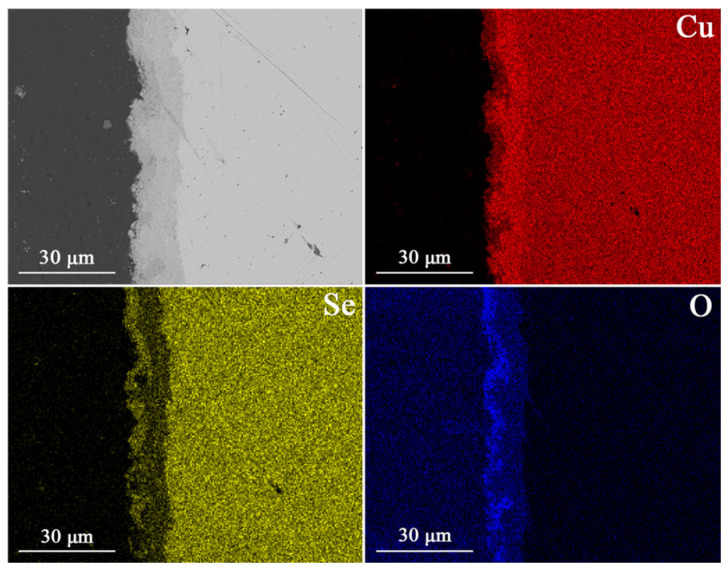
SEM photographs of selected cross-section with respective maps of element distribution for Cu_2_Se sample for oxidized at 673 K for 1 h in air.

**Figure 2:**
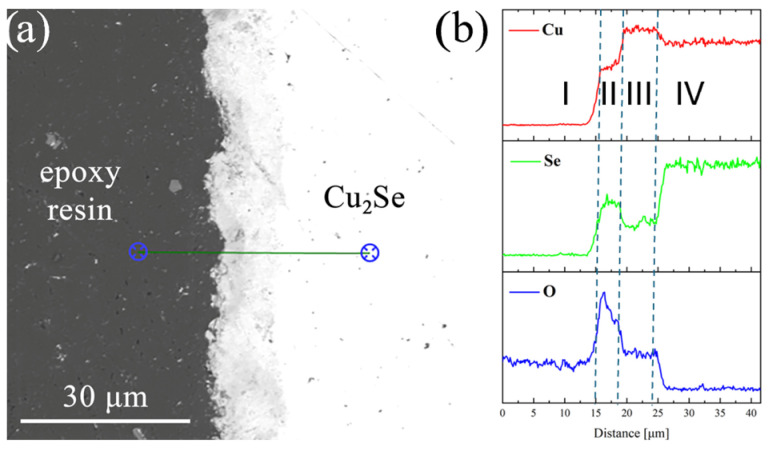
(**a**) SEM images with (**b**) linear analyses of the chemical composition for Cu_2_Se samples oxidized at 673 K for 1 h in air (I—epoxy resin, II—CuO, III—Cu_2_O, IV—Cu_2_Se).

Figure 1 and Figure 2 show SEM images of the Cu_2_Se sample annealed for 1 h at *T* = 673 K. Point and line chemical composition analyses using SEM–EDS indicate the presence of copper oxides, Cu_2_O and CuO, on the Cu_2_Se surface. Specifically, copper(I) oxide (Cu_2_O) grows directly on the Cu_2_Se surface, followed by copper(II) oxide (CuO). This is consistent with the growth mechanism described by Taskinen et al. [38].

**Figure 3:**
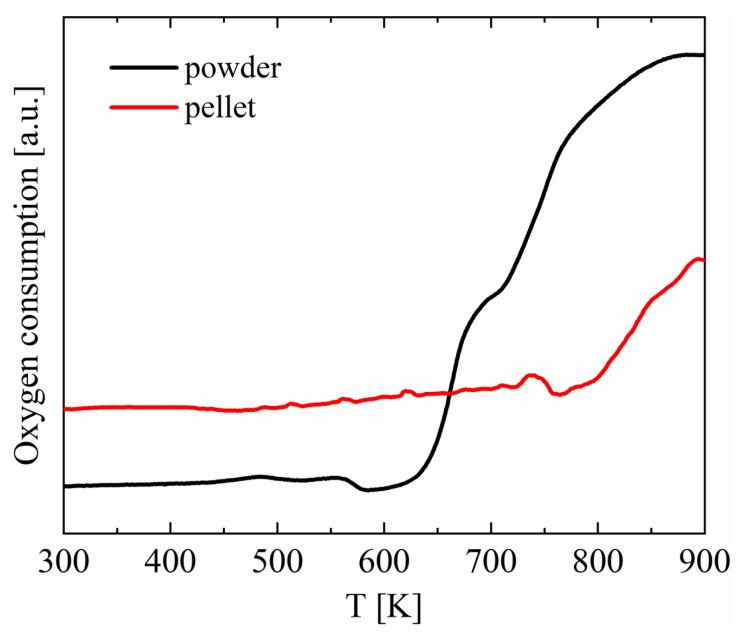
TPO_x_ measurements for pellets and powder samples of Cu_2_Se.

To thoroughly investigate the oxidation process occurring on the Cu_2_Se surface, TPO_x_ measurements were conducted (Figure 3). The analysis of results for the sintered pellet shows significantly smaller changes related to oxygen consumption compared to the powdered pellet, with a distinct increase beginning at approximately 800 K. For powdered pellets, where the free surface area is considerably larger, oxidation processes are clearly visible, and significant changes in oxygen consumption start at around 650 K. This result is consistent with the SEM studies, where a distinct layer of copper oxides is visible in the analyses as early as 673 K (Figure 1). Additionally, Xiao et al. [44] demonstrated the presence of Cu_2_O during temperature-dependent XRD studies starting from 673 K (it was not visible on diffractograms at lower temperatures), which they attributed to low vacuum during the high-temperature XRD testing. The above results indicate that prolonged exposure of Cu_2_Se-based materials to air, even at 673 K, can lead to significant material degradation.

**Figure 4:**
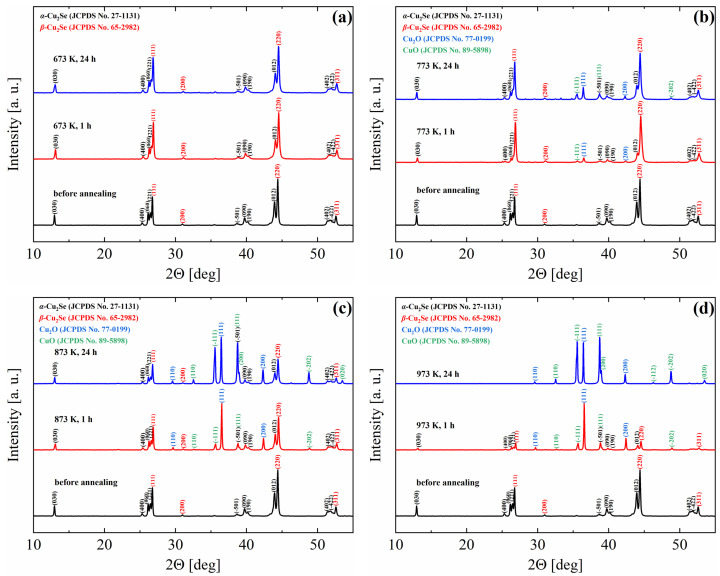
XRD patterns of powdered sinters after oxidation (**a**) at 673 K, (**b**) 773 K, (**c**) 873 K and (**d**) 973 for 1 h and 24 h measurements for pellets and powder samples of Cu_2_Se.

Figure 4a–d presents the *X*-ray diffraction (XRD) results for Cu_2_Se samples after SPS and subsequent annealing at 673, 773, 873, and 973 K in an air atmosphere for powdered pellets. XRD analysis of the SPS-sintered samples before annealing (Figure 4) revealed the presence of both *α*-Cu_2_Se and *β*-Cu_2_Se phases. This finding aligns with the results from other studies, where copper(I) selenide was sintered using SPS [45,46] hot pressing [47], or arc-melting [48]. For samples annealed at 673 K (Figure 4b) even after 24 h of annealing, no phases other than *α*-Cu_2_Se and *β*-Cu_2_Se were observed. This suggests a very small quantity of copper oxides (which were confirmed by SEM–EDS analysis on the sample surfaces; see Figure 1 and Figure 2 within the bulk material, falling below the detection limit of the XRD method. In contrast, for samples annealed at 773 K, both Cu_2_O and CuO oxides are clearly visible, and their quantity increases with annealing time (Figure 4b). The amount of oxides drastically increases during Cu_2_Se annealing at 873 K and 973 K, and their quantity continues to rise with annealing time (Figure 4c,d). It is important to note that in the sample annealed at 973 K for 24 h in air, only copper oxides (Cu_2_O and CuO) are observed, while no reflections originating from the Cu_2_Se phase are visible (Figure 4d). This result indicates very rapid degradation of Cu_2_Se within the temperature range where the highest *ZT* values are typically observed (for doped Cu_2_Se samples or those with nano-inclusions). This degradation effectively precludes the use of this material in thermoelectric generators operating in an air atmosphere, and highlights the necessity of applying appropriate protective layers to safeguard the material from degradation.

**Figure 5:**
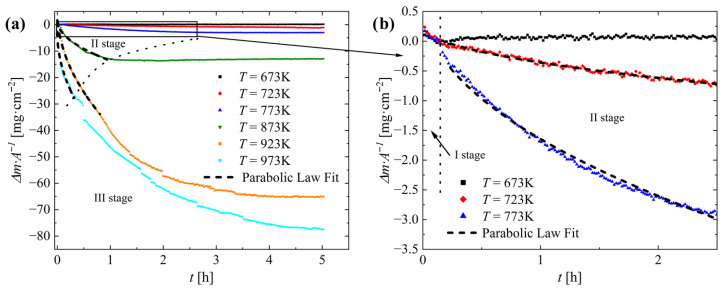
(**a**) Change in mass per unit surface as a function of time for Cu_2_Se sinter samples heated in air at different temperatures and (**b**) enlarged fragment of the graph for selected samples in the range *T* = 673–773 K and *t* = 0–2.5 h.

To investigate the oxidation kinetics of Cu_2_Se and gain a better understanding of the reaction mechanism, we conducted thermogravimetric isothermal oxidation measurements. Previous studies [38] and our own SEM, XRD and TPO measurements have shown that several parallel processes occur during the oxidation of Cu_2_Se. These include the oxidation of Cu_2_Se to copper oxides, selenium oxide, and copper(I) selenate(VI); the sublimation of selenium(IV) oxide; and further reactions of the resulting oxidation products, such as the oxidation of Cu_2_O to CuO or the decomposition of Cu_2_SeO_4_ to CuO ⋅ CuSeO_3_. However, previous studies have not revealed the exact reaction mechanism(s) in our studied temperature range, nor have they identified which diffusion pathways are dominant. An examination of the mass-versus-time dependence for the isothermal oxidation of Cu_2_Se (Figure 5) shows that the mechanism is different at temperatures below 723 K, where mass initially decreases and then begins to increase during oxidation, compared to the 723−973 K range. For measurements performed at temperatures above 673 K, most samples exhibit an increasing mass loss over time, with the exception of samples oxidized between 773 K and 923 K, where the mass begins to increase slightly after a certain period. The results of isothermal oxidation also indicate that the reaction mechanism changes with the degree of conversion, and the oxidation process can be roughly divided into three stages. In the first stage, reaction initiation/incubation occurs on the sample surface, during which copper oxidizes to copper oxide (CuO) and selenium oxidizes to SeO_2_, which sublimates almost immediately. The resulting copper oxide grains grow, covering an increasing portion of the sample surface. This stage concludes when the entire surface is covered by a dense layer of reaction products. The oxidation rate (measured as mass loss) during this stage may depend on the reaction surface area, the sublimation rate of SeO_2_, or other processes. In any case, it is close to a linear dependence on time for samples oxidized at 673−773 K. Because this is also the period during which the sample undergoes nonlinear heating to the experimental temperature, a kinetic analysis of the reaction is not practically feasible. In the next stage, the mass-versus-time dependence becomes parabolic, indicating that the oxidation process is controlled by the diffusion of reactants through the scale layer. The third stage depends on the oxidation temperature. At temperatures below 773 K, after a certain degree of conversion is reached, the mass loss rate slows down. This may be caused by the equalization of the SeO_2_ sublimation rate and the Cu_2_Se oxidation rate, the loss of contact between the scale layer and the unoxidized substrate, or the inhibition of the reaction by the formation of a product layer that acts as a diffusion barrier for Se^4+^/SeO_2_. At temperatures of 923 K and higher, an increase in the reaction rate is observed, and the mass loss-versus-time dependence becomes more linear, or a sudden, stepped drop in mass is observed, which can be attributed to the cracking and spalling of the scale, as observed on the samples after the experiments. A more detailed understanding of the reaction mechanisms at each stage would require more in-depth studies, which were not the objective of this publication.

**Figure 6:**
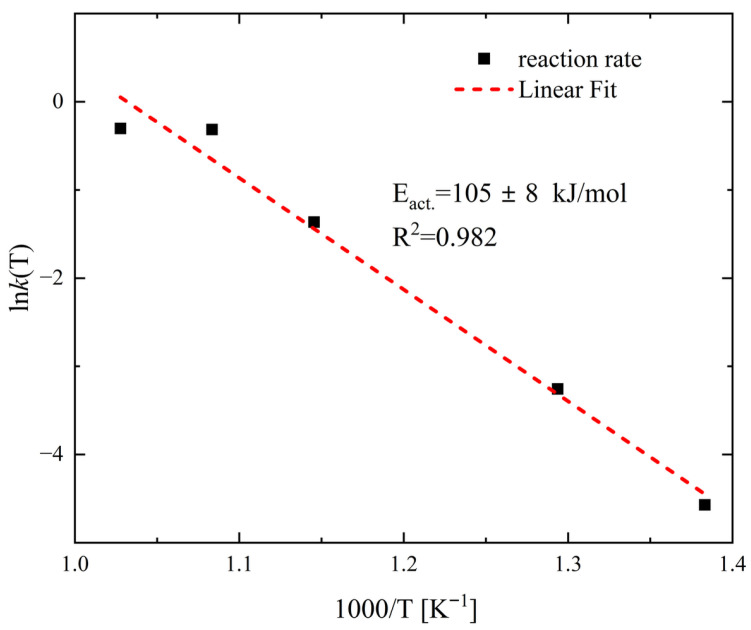
Temperature dependence of the parabolic rate constant of Cu_2_Se oxidation process presented in Arrhenius plot.

For the TG curves corresponding to the second oxidation stage, the mass loss-versus-time dependence was fitted to a parabolic law. The parabolic rate constants obtained in this way were plotted on an Arrhenius graph (Figure 6). In the temperature range of 723 K to 923 K, the points form a linear relationship. The activation energy value calculated from the slope of the line is 105 kJ·mol^−1^ ± 8 kJ/mol. Given that this stage corresponds to the growth of a dense layer of copper oxides on the Cu_2_Se surface and the reaction kinetics are consistent with a mechanism controlled by the diffusion of a reactant through a product layer of increasing thickness, the rate-controlling process should be sought among the diffusion processes through the copper oxides. Studies of the defect structure of copper oxides and their oxidation mechanisms show that mass transport is primarily due to the diffusion of copper ions [49,50]. Depending on the copper oxide, its purity, thickness, and oxidation conditions, the activation energy of oxidation associated with copper ion migration ranges from 30 to 170 kJ·mol^−1^ [49,50,51,52,53]. A review of the literature also shows that the activation energies for the diffusion of copper ions in Cu_2_O and CuO are similar in comparable temperature ranges, e.g., 164 ± 7 kJ·mol^−1^ at 973–1123 K for CuO [52], 151.04 kJ·mol^−1^ at 1073–1323 K for Cu_2_O [49], and 147 kJ·mol^−1^ at 973–1273 K for CuO [50]. The closest activation energy value to our measured value of approximately 111 kJ·mol^−1^ can be found for the oxidation of copper in the temperature range of 873–1073 K [51], which is attributed to the growth of a Cu_2_O layer via the diffusion of copper through grains and grain boundaries. SEM analysis and a linear composition analysis of the resulting scale show that selenium does not accumulate at any of the interfaces between the sample and the growing copper oxide layers, but it is present to some extent within the oxide layers. The copper selenate formed in small quantities during oxidation at the interface between Cu_2_Se and the Cu_2_O layer was not visible in our measurements. The lack of selenium accumulation, whether as SeO_2_ or copper selenate, indicates that the outward diffusion process of selenium and its sublimation must be faster than the outward diffusion of copper through the oxide layer, and thus is likely not the rate-limiting step for the oxidation reaction. Therefore, based on the collected results, we propose that the observed activation energy for the second stage of oxidation can be attributed to the outward diffusion of copper through the grains and grain boundaries of the copper oxides, and we can identify this process as the rate-limiting step.

## 4. Conclusions

Research on the stability of copper(I) selenide (Cu_2_Se) in an air atmosphere has revealed significant limitations that prevent its direct application in thermoelectric generators (TEGs), especially within the temperature range where materials based on it exhibit the highest *ZT* parameter values (873–973 K). The material’s degradation proceeds in two ways: on one hand, through the formation of copper oxides (Cu_2_O and CuO), as shown by SEM–EDS and XRD studies, and equally importantly, through the sublimation of selenium oxide (SeO_2_), as shown by thermogravimetric analysis. Isothermal oxidation measurements confirmed that the oxidation process is complex and, after an initial stage, the dependence of mass change on time is parabolic. This indicates that the process is controlled by the outward diffusion of copper through the copper oxide layer. The results obtained in this work clearly indicate that to utilize the excellent thermoelectric properties of Cu_2_Se-based materials in practical TEG applications in an air atmosphere, protective layers must be used to prevent their degradation in an oxidizing atmosphere. As a continuation of the presented research, we plan to undertake new studies in the near future, focused on developing effective protective coatings for Cu_2_Se. These studies will be based on our previous results concerning pure and modified protective layers of silicon oxycarbide (SiOC) [41,42,54].

## Data Availability

The original contributions presented in this study are included in the article/Supplementary Material. Further inquiries can be directed to the corresponding author.

## Supplementary Materials

The following supporting information can be downloaded at: https://www.mdpi.com/article/10.3390/ma18174152/s1, Figure S1: Schematic diagram of the setup used for isothermal thermogravimetric oxidation study.
