# Supplementary material for: Investigating the Stability of Cu2Se Superionic Thermoelectric Material in Air Atmosphere
Source: Materials (Basel). 2025 Sep 4;18(17):4152. doi: 10.3390/ma18174152 (PMC12430787; doi:10.3390/ma18174152)
Supplement: Supplementary file 1 [file materials-18-04152-s001.zip › materials-3833212-supplementary.pdf]

Supplementary material

Investigating the stability of Cu_2Se superionic thermoelectric material in air atmosphere

Paweł Nieroda [1], Małgorzata Rudnik [1], Marzena Mitoraj-Królikowska [1], Ewa Drożdż [1], Dawid Kozień [1], Juliusz Leszczyński [1], Andrzej Koleżyński [1,*]

[1] Faculty of Materials Science and Ceramics, AGH University of Krakow, al. A. Mickiewicza 30, 30-059 Krakow, Poland; pnieroda@agh.edu.pl (P.N), malrud@student.agh.edu.pl (M.R), mmitoraj@agh.edu.pl (M.M), edrozdz@agh.edu.pl (E.D), jleszczy@agh.edu.pl (J.L.), kozien@agh.edu.pl (D.K), andrzej.kolezynski@agh.edu.pl (A.K.)

* Correspondence: andrzej.kolezynski@agh.edu.pl

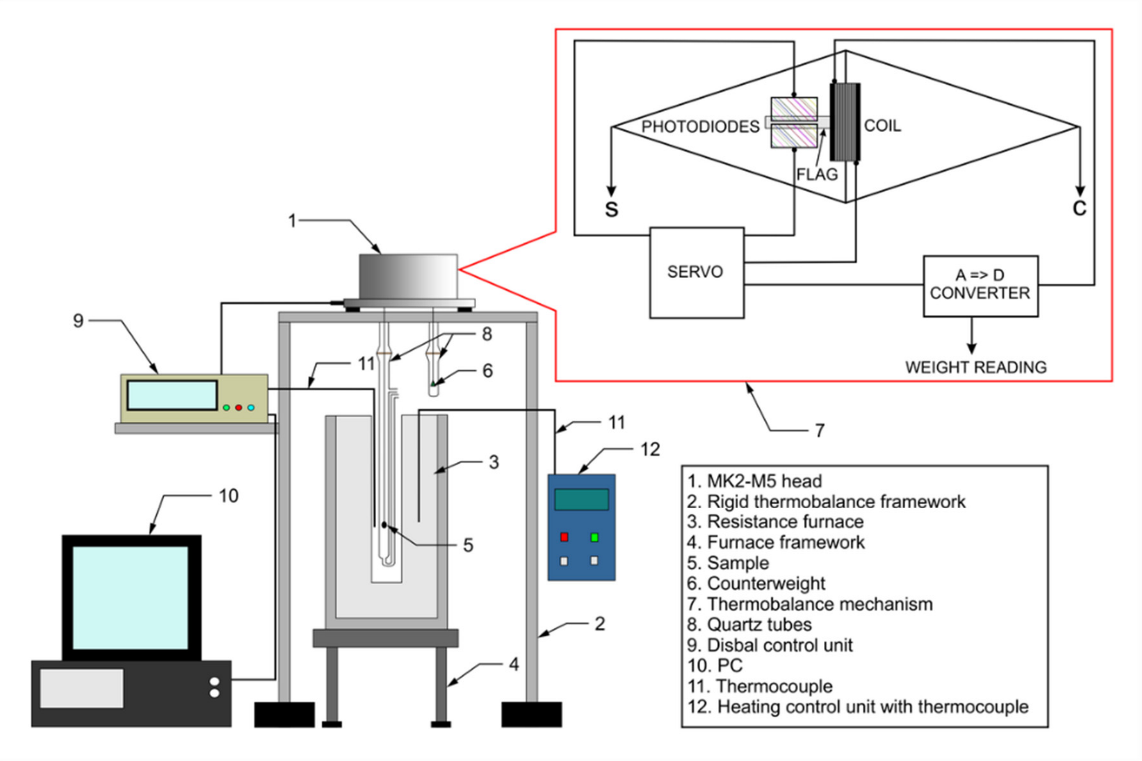

Figure S1. Schematic diagram of the setup used for isothermal thermogravimetric oxidation study.
